# UmuDAb: An Error-Prone Polymerase Accessory Homolog Whose N-Terminal Domain Is Required for Repression of DNA Damage Inducible Gene Expression in *Acinetobacter baylyi*

**DOI:** 10.1371/journal.pone.0152013

**Published:** 2016-03-24

**Authors:** Travis A. Witkowski, Alison N. Grice, DeAnna B. Stinnett, Whitney K. Wells, Megan A. Peterson, Janelle M. Hare

**Affiliations:** Department of Biology and Chemistry, Morehead State University, Morehead, KY, United States of America; Florida International University Bimolecular Sciences Institute, UNITED STATES

## Abstract

In many bacteria, the DNA damage response induces genes (SOS genes) that were repressed by LexA. LexA represses transcription by binding to SOS promoters via a helix-turn-helix motif in its N-terminal domain (NTD). Upon DNA damage, LexA cleaves itself and allows induction of transcription. In *Acinetobacter baumannii* and *Acinetobacter baylyi*, multiple genes are induced by DNA damage, and although the *Acinetobacter* genus lacks LexA, a homolog of the error-prone polymerase subunit UmuD, called UmuDAb, regulates some DNA damage-induced genes. The mechanism of UmuDAb regulation has not been determined. We constructed UmuDAb mutant strains of *A*. *baylyi* to test whether UmuDAb mediates gene regulation through LexA-like repressor actions consisting of relief of repression through self-cleavage after DNA damage. Real-time quantitative PCR experiments in both a null *umuDAb* mutant and an NTD mutant showed that the DNA damage-inducible, UmuDAb-regulated gene *ddrR* was highly expressed even in the absence of DNA damage. Protein modeling identified a potential LexA-like helix-turn-helix structure in the UmuDAb NTD, which when disrupted, also relieved *ddrR* and *umuDAb* repression under non-inducing conditions. Mutations in a putative SOS box in the shared *umuDAb-ddrR* promoter region similarly relieved these genes’ repression under non-inducing conditions. Conversely, cells possessing a cleavage-deficient UmuDAb were unable to induce gene expression after MMC-mediated DNA damage. This evidence of a UmuDAb repressor mechanism was contrasted with the failure of *umuDAb* to complement an *Escherichia coli umuD* mutant for UmuD error-prone DNA replication activity. Similarly, *A*. *baumannii* null *umuDAb* mutant cells did not have a reduced UmuDˊ_2_UmuC-mediated mutation rate after DNA damage, suggesting that although this UmuDAb protein may have evolved from a *umuDC* operon in this genus, it now performs a LexA-like repressor function for a sub-set of DNA damage-induced genes.

## Introduction

Bacteria use many genes to sense, respond to, and activate repair of damaged DNA. Such a DNA damage repair regulon is typically maintained under LexA repression until DNA damage triggers relief of that repression [1,2] and allows transcription. In the absence of DNA damage, LexA recognizes and binds to a conserved operator (the SOS box) in the promoters of SOS (DNA damage-inducible) genes [3], repressing their expression. An unconventional winged helix-turn-helix (wHTH) motif [4] in the LexA N-terminal domain (NTD) facilitates its binding as a dimer [5] to the DNA backbone of the palindromic SOS box. When DNA damage occurs, which can be caused by UV radiation, chemicals such as mitomycin C (MMC), or certain antibiotics, the recombination protein RecA is activated, and facilitates the intramolecular self-cleavage of the LexA dimer [6]. The conformational change in the protein facilitates its dissociation from promoters [1], and leads to the increased expression of genes that sense, regulate, repair and replicate DNA in the damaged cell [7,8].

In the Gram-negative, proteobacterial bacterial genus *Acinetobacter*, regulons of DNA damage-inducible genes have been identified in both the non-pathogenic model organism, *Acinetobacter baylyi* strain ADP1, as well as the opportunistic, multi-drug resistant pathogen, *Acinetobacter baumannii* [9,10]. However, all attempts to identify a *lexA* homolog in this genus have been fruitless [11,12], raising the question of how target genes are repressed and induced in response to DNA damage. Other deviations in *Acinetobacter* spp. from the general proteobacterial model include the RecA-independent induction of *recA* [9,13,14], which may indicate a profoundly different DNA damage response mechanism than that of other proteobacteria. Alternately, a protein lacking homology to LexA may assume LexA-like repressor functions in this genus.

Other investigations of the *Acinetobacter* DNA damage response mechanism have focused on the *umuDC* SOS operon. In the *E*. *coli* SOS response, induced but uncleaved UmuD initially conducts DNA checkpoint functions [15]. After self-cleavage, the UmuDˊ protein associates with UmuC to form the UmuDˊ_2_UmuC type V polymerase that conducts error-prone translesion DNA synthesis [16,17]. However, in the multiple *A*. *baumannii* strains that have been sequenced, each strain possesses multiple *umuDC* operons [14,18]. In *A*. *baumannii* these operons collectively cause increased mutagenesis and thus antibiotic resistance after DNA damage [14,19]. However, an additional *umuD* allele, termed *umuDAb*, is also present throughout the members of the *Acinetobacter* genus [18].

The *Acinetobacter* UmuDAb differs from UmuD (both its own and those found throughout the proteobacteria) because it encodes an additional 59 amino acid NTD (and is the same size as LexA [11]), and regulates the expression of DNA damage responsive genes, such as itself and the divergently transcribed *ddrR* gene located adjacent to *umuDAb* [9–11]. Specifically, its regulation seems to involve repression of only a limited set of genes in *A*. *baumannii*: itself, all *umuDC* operons and homologs, and *ddrR* [9,10]. UmuDAb, like UmuD, LexA, and many bacteriophage repressors, conducts RecA-mediated self-cleavage at a conserved site (Ala83-Gly84 for UmuDAb) using enzymatic Ser119 and Lys156 residues in its C-terminal domain (CTD; here defined by the amino acids 83–203, including, and C-terminal to, the cleavage site) [20]. This can occur in *E*. *coli* when UmuDAb is expressed, where cleavage after DNA damage requires the action of *E*. *coli* RecA [20]. However, UmuDAb possesses chimeric features of self-cleavage: it cleaves slowly, like UmuD, but only intramolecularly, like LexA and bacteriophage repressors [20]. These similarities to LexA suggests that its mechanism of regulating target gene expression might involve an analogous process.

Arguing against a strictly regulatory role for UmuDAb, however, is the observation that BLASTp alignments of LexA and UmuDAb do not align these proteins’ N-terminal ~78 amino acids. Additionally, conserved domain searches do not reveal any helix-turn-helix (HTH) motif structure with which UmuDAb might repress gene expression. UmuDAb is also more similar to UmuD (46% identity over 134 C-terminal amino acids) than to LexA (37% identity over 115 C-terminal amino acids). Furthermore, in at least five *Acinetobacter* species, and multiple strains, the *umuDAb* gene is part of a complete *umuDAb-umuC* operon (or incomplete; in *A*. *baylyi* strain ADP1 the *umuC* gene encoded downstream of *umuDAb* is truncated) [11]. This suggests that *umuDAb* may have evolved from a *umuDC* operon and therefore might function, like UmuD, as an error-prone polymerase accessory. An observation of reduced rifampin resistance in an *A*. *baumannii umuDAb* mutant suggests this possibility [10].

Although UmuDAb appears to regulate DNA damage-inducible genes like a repressor [9,10], and the *A*. *baumannii* UmuDAb can bind to the putative promoter region shared by *umuDAb* and *ddrR* [10], no experiments have been conducted to test whether its DNA binding and self-cleavage activities [20] are involved in its regulatory function. Our objective was to determine if, and how, the NTD and CTD of UmuDAb were required for its regulation of DNA damage-inducible genes. The LexA model predicts that transcriptional repression mediated by promoter binding is relieved by UmuDAb self-cleavage after DNA damage, and thus implies a role for both the UmuDAb NTD and CTD, as well as a temporal order of their action. The expression levels of *A*. *baylyi* strain ADP1 DNA damage-inducible genes that are known to be repressed in *A*. *baumannii* [9,10] were measured with real-time quantitative PCR (RT-qPCR) in a variety of *umuDAb* mutant strains after exposure to MMC. These experiments revealed a requirement for the NTD (and a potential HTH motif therein) in the repression of DNA damage-inducible target genes, as well as the CTD cleavage site in allowing target gene induction after DNA damage. Mutation of the operator of target gene promoters also abolished repression of gene expression under non-inducing conditions. These experiments suggest a regulatory mechanism of action of UmuDAb that closely resembles LexA, even though the NTDs of these proteins do not share a high degree of amino acid identity.

We additionally sought to distinguish between regulatory (LexA-like) and potential error prone polymerase (UmuD-like) actions of UmuDAb. The *Acinetobacter umuDAb* was unable to complement an *E*. *coli umuD* null mutant for DNA damage-induced mutagenesis, and a *umuDAb* null mutant of *A*. *baumannii* did not display reduced rifampin resistance in the absence of DNA damage, suggesting that the action of UmuDAb is regulatory, albeit for only a specialized sub-set of DNA damage inducible genes in this genus.

## Materials and Methods

### Bacterial strains and growth conditions

*Acinetobacter baylyi* ADP1 and *A*. *baumannii* ATCC 17978 strains were grown in minimal media with 10 mM succinate at 37°C. *Escherichia coli* AB1157 and 315 (AB1157 *ΔumuD772*::*kan*) were grown in LB broth at 37°C.

For RT-qPCR analyses performed as previously described [9], all *A*. *baylyi* ADP1-derived strains (see Table 1) were grown in a 3 ml overnight culture at 37°C at 250 rpm in minimal media with 10 mM succinate. Overnight cultures were diluted 1:25 into 5 mL fresh media and grown with shaking for two hours before the culture was split, with 2 μg/mL MMC added to one culture. Further incubation for three hours induced gene expression, in keeping with previous studies by the authors **[9]** and others **[10]** when using mitomycin C as the DNA damaging agent in gene expression studies.

**Table 1:** Bacterial strains and plasmids used in this study.

Strain or plasmid	Description or genotype	Source/Reference
AB1157	*E*. *coli* wild type	P. Beuning
315	*E*. *coli* AB1157 Δ*umuD*772::kan; Kan^R^	P. Beuning
ADP1	*A*. *baylyi* wild type	This study
ACIAD2729	*A*. *baylyi* ADP1 Δ*umuDAb*::*tdk-kanR*; Kan^R^	[21]
ACIAD2730	*A*. *baylyi* ADP1 Δ*ddrR*::*tdk-kanR*; Kan^R^	[21]
JHTW1	*A*. *baylyi* ADP1 *umuDAbΔ2–59*	This study
JHKW1	*A*. *baylyi* ADP1 *umuDAbΔ2–83*	This study
JHDS1	*A*. *baylyi* ADP1 *umuDAb A83Y*	This study
JHTW2	*A*. *baylyi* ADP1 *umuDAb K40P R41P*	This study
JHDT1	*A*. *baylyi* ADP1 *umuDAb K40P R41P A83Y*	This study
JHMP1	*A*. *baylyi* ADP1 *umuDAb E24K*	This study
JH100	*A*. *baylyi* ADP1 with an eight bp substitution mutation in *umuDAb*-*ddrR* promoter; see Fig 4	This study
JH101	*A*. *baylyi* ADP1 with an eight bp substitution mutation in *umuDAb*-*ddrR* promoter; see Fig 4	This study
JH102	*A*. *baylyi* ADP1 with a three bp substitution mutation in *umuDAb*-*ddrR* promoter; see Fig 4	This study
JH103	*A*. *baylyi* ADP1 with a three bp substitution mutation in *umuDAb*-*ddrR* promoter; see Fig 4	This study
JH104	*A*. *baylyi* ADP1 with six bp substitution mutation in *umuDAb*-*ddrR* promoter; see Fig 4	This study
17978	*A*. *baumannii* ATCC 17978; wild type	ATCC
17978 Δ*umuDAb*	*A*. *baumannii* ATCC 17978 Δ*umuDAb* (A1S_1389)	[9]
17978 *umuDAbˊ*	*A*. *baumannii* ATCC 17978 *umuDAbˊ*; TetR	This study
pGEM®-T Easy	TA cloning vector; Amp^R^	Promega
pTW1	*A*. *baylyi* genomic DNA containing *umuDAb* and *ddrR* genes, cloned into pGEM®-T Easy	This study
pIX3.0	Expression vector; Amp^R^	Qiagen
pIX2b	pIX2; pIX3.0 carrying *A*. *baylyi* ADP1 *umuDAb*; Amp^R^	[20]
pIX2AtoY	pIX2 bearing site directed mutation of *umuDAb* codon 83 (GCT) to TAT, yielding A83Y mutation of *umuDAb*; Amp^R^	[20]
pIX2bˊ	pIX3.0 carrying *A*. *baylyi* ADP1 *umuDAbˊ*; Amp^R^	This study
pIXUDEC	pIX3.0 carrying *E*. *coli umuD*; Amp^R^	This study

### Mutant strain construction

All strains of ADP1 possessing mutations in the chromosomal copy of *umuDAb* (ACIAD2729) or its promoter region were constructed by transforming a linear PCR product containing the mutant, unmarked allele into the *ΔumuDAb*::*tdk-kanR* strain ACIAD2729. Recombinant mutant strains were counter-selected for allelic replacement of the *tdk*-*kanR* cassette on azidothymidine (200 μg/mL) agar medium. Azidothymidine-resistant colonies were screened for loss of the kanamycin resistance phenotype. The resultant chromosomal mutations were confirmed by PCR analysis, sequencing of *umuDAb*, and Western blot analysis indicating UmuDAb production.

The mutant strains JHTW1 (*umuDAbΔ2–59*) and JHKW1 (*umuDAbΔ2–83*) were constructed by deleting, in frame, part of the *umuDAb* coding region (58 codons or 82 codons, respectively) in an inverse PCR amplification performed on pTW1, using primers listed in Table 2. pTW1 contains a 2,652 bp *A*. *baylyi* genomic DNA fragment, PCR amplified with primers CL-N and ToJH1Hd, ligated into the Promega vector pGEM®-T Easy. The resulting inverse amplified PCR products were blunted using Epicentre End-It™ DNA End-Repair Kit and re-ligated. The mutant *umuDAb* allele and flanking regions were PCR-amplified using CL-N and ToJH1Hd, purified, then transformed into ACIAD2729.

**Table 2:** Oligonucleotide primers used in this study.

Primer name	Purpose	Primer sequence
CL-N	Amplification from pTW1 to construct mutant strains	ATAGTGTTGGTATGATGCG
ToJH1Hd	Amplification from pTW1 to construct mutant strains	GACAAGCTTAGAGTTGAATA
TJ2b	JHTW1 (*umuDAbΔ2–59*) construction	GTCCAGGCGATACAGCCCAA
TJ1	JHKW1 (*umuDAbΔ2–83*) construction	CATCAGCCCTCCTAACACAA
umuD’For	JHKW1 (*umuDAbΔ2–83*) construction	GGTTTGCCATCACCTGCACA
CL0	JHDS1 (*umuDAb A83Y)* construction	AGCCAACTAAAGTCATTCG
CL10	JHDS1 (*umuDAb A83Y)* construction	TTTTCATCCGCCTAAAG
CLK	JHDS1 (*umuDAb A83Y)* construction	TAACGCATAGGTTTCAGATTG
a118c-a119c_g122c_For	JHTW2 (*umuDAb K40P R41P*) construction	CGAGTGCCAGAATCTCAGGTTGCTTTTATTCCGCCTTGGCTTTTAGATAAC
a118c-a119c_g122c_Rev	JHTW2 (*umuDAb K40P R41P*) construction	GTTATCTAAAAGCCAAGGCGGAATAAAAGCAACCTGAGATTCTGGCACTCG
DSumuDfor	JHDT1 (*umuDAb K40P R41P A83Y*) construction	TCTTATTGATTTTAATTCGGC
CL-4	JHDT1 (*umuDAb K40P R41P A83Y*) construction	CCTGCTTATGCAATGACAG
ExtraUDRev	JHDT1 (*umuDAb K40P R41P A83Y*) construction	GCCTGGACTTTCAGTGC
To81Rev	JHDT1 (*umuDAb K40P R41P A83Y*) construction	CTGAACGTATTTGATTGAGC
g70a	JHMP1 (*umuDAb E24K*) construction	CTGGACGTAAGGCCAAATACCAAAAGCCAACTAAAGT
g70	JHMP1 (*umuDAb E24K*) construction	ACTTTAGTTGGCTTTTGGTATTTGGCCTTACGTCCAG
SDMFirF	JH100 promoter mutation construction	TTATCGTGCGTCTCTCAACGTTTGTAACGA
SDMFirR	JH100 promoter mutation construction	TCGTTACAAACGTTGAGAGACGCACGATAA
SDMPromIR2For	JH101 promoter mutation construction	TGAATTTGTAACGATGAGCTAGCAGATTATTTTAACTTG
SDMPromIR2Rev	JH101 promoter mutation construction	CAAGTTAAAATAATCTGCTAGCTCATCGTTACAAATTCA
SDMFir1AFor	JH102 promoter mutation construction	ATTATCGTGCGTCTCTTTGAATTTGTAACGA
SDMFir1ARev	JH102 promoter mutation construction	TCGTTACAAATTCAAAGAGACGCACGATAAT
SDMPstPromFor	JH103 promoter mutation construction	CTTGAATCTGCAGCGATTTCAAGTTAGATT
SDMPstPromRev	JH103 promoter mutation construction	GAAATCGCTGCAGATTCAAGTTGACGCACGATAA
SDMMidEndFor	JH104 promoter mutation construction	AATAATCTAACTTGGCGCACTTACAAATCAAGT
SDMMidEndRev	JH104 promoter mutation construction	ACTTGAATTTGTAAGTGCGCCAAGTTAGATTATT
2731RTFor2	JH100-JH104 promoter mutation construction	ACGATGGGCATGGATGAAGTGG
17TJ1analog	17978 *umuDAbˊ* construction	CATAATCGCCTCCATTTCAC
17TJ1analogFor	17978 *umuDAbˊ* construction	GGTTTCCCATCACCAGC
E.coliNHisumuDS	*E*. *coli umuD* expression	ACCCACGCGCATGTCGTAAAAAGCACCCAATTGTTTATCAAGCCTGC
E.coliNHisumuDAS	*E*. *coli umuD* expression	CTTGGTTAGTTAGTTATTATCAGCGCATCGCCTTAACGA
ddrR#RTFor	RT-qPCR on *ddrR*	ATACCGAACAAGCCGAGCAT
ddrR#2RTRev	RT-qPCR on *ddrR*	AGGCATGACTAAAGCCAGCA
umuDAb#RTFor	RT-qPCR on *umuDAb*	GGAGCATGTCGAGCAGAGTC
umuDAb#2RTRev	RT-qPCR on *umuDAb*; also inJH100-JH104 promoter mutation construction	TCACCTGCTTTGGCCGTAAT
0445RTFor	RT-qPCR on *gst* (ACIAD0445)	ACCTGTACTCACTGATGGCG
0445RTRev	RT-qPCR on *gst* (ACIAD0445)	ACAGACCTCGTTTCGGATCA
ADP0724RTFor	RT-qPCR on *nrdA*	ATGACCGTCGTCGTACTCAC
ADP0724RTRev	RT-qPCR on *nrdA*	GCTGTGCAAATTCTTCGCCA
16SrRNA#RTFor	RT-qPCR reference primer	CCACACTGGGACTGAGACAC
16SrRNA#2RTRev	RT-qPCR reference primer	AACCAGGTAAGCCTCCTCCT

The *umuDAb K40P R41P* site directed mutation in strain JHTW2, and the *umuDAb E24K* site directed mutation in JHMP1, was constructed using the QuikChange II XL Site-Directed Mutagenesis Kit and mutagenic primers listed in Table 2 with pTW1 as template for the mutagenic reactions.

To make the various mutations in the *ddrR*-*umuDAb* promoter region of ADP1 in strains JH100-JH104, PCR products were amplified from ADP1 genomic DNA, using the mutagenic primers indicated in Table 2. One mutagenic primer was paired with either of the outside primers 2731RTFor2 and umuDAb#2RTRev, which flank the promoter region. Splice-overlap-extension (SOE) PCR using these two products and primers 2731RTFor2 and umuDAb#2RTRev in a second PCR reaction was used to construct a linear template for recombination into the ADP1 chromosome.

To construct the *umuDAb A83Y* mutation in JHDS1, a three piece SOE strategy was employed, with amplification from (i) ADP1 genomic DNA using primers CL-N and CL-10, (ii) the pIX2AtoY plasmid containing the previously constructed *umuDAb A83Y* mutation (20), using primers CL-K and CL-0, and (iii) plasmid pJH1 (11) using primers ExtraUDRev and To81Rev. Assembly of these three PCR products in SOE PCR was performed using primers CL-N and To81Rev.

To construct the *umuDAb K40P R41P A83Y* mutation in JHDT1, a two piece SOE strategy was employed, with amplification from (i) the *umuDAb K40P R41P* mutant JHTW2, using primers To81Rev and ExtraUDRev, and (ii) the *umuDAb A83Y* mutant JHDS1, using primers DSumuDfor and CL-4. These two PCR products served as template in a SOE PCR amplification with primers To81Rev and CL-4.

These SOE PCR products were transformed into ACIAD2730 (to make strains JH100-JH104) or ACIAD2729 (to make all other strains). Recombinants were counter-selected for on azidothymidine plates, screened by PCR and for loss of kanamycin resistance, and sequenced, as described above.

To construct a strain of *A*. *baumannii* ATCC 17978 expressing only the cleaved form of UmuDAb (UmuDAbˊ), inverse PCR with primers 17TJ1analog and 17TJ1analogFor was performed on a plasmid carrying this species’ *umuDAb* chromosomal region to delete codons for amino acids 2–83. The tetracycline resistance cassette of pBR322 was amplified with PCR and cloned into an *Nru*I site located 99 bp downstream of the end of *umuDAb*. Transformation of this plasmid into the previously constructed 17978 *ΔumuDAb* strain [9] followed by selection on tetracycline-containing plates introduced this mutant *umuDAb* allele into cells. Genetic construction of this strain was confirmed with PCR analyses.

### Gene expression experiments

RT-qPCR experiments were conducted essentially as described previously (9) from triplicate biological samples grown in minimal medium plus 10 mM succinate and induced for three hours with 2 μg/mL MMC. The gene expression of two strains was measured on each 96-well RT-qPCR plate, comparing expression from reference primers 16SrRNA#RTFor and 16SrRNA#2RTRev to the test primers for each gene of interest (*ddrR* (*ACIAD2730*), *umuDAb* (*ACIAD2729*), *gst* (*ACIAD0445*), and *ndrA* (*ACIAD0724*); see Table 2). Primer efficiency and no-template controls were performed to validate the process (S1 Table), with all efficiencies of target and reference genes having similar (within 3%) efficiencies, as recommended [22]. Transcriptional changes (induction due to DNA damage) were calculated using the 2^−ΔΔCT^ method (22) and GraphPad InStat was used to conduct all statistical analyses, including ANOVA and t-tests.

### DNA damage-induced mutations

Evaluating increased resistance to rifampin after UV exposure (here, 200 J/m^2^), as a measure of error-prone polymerase activity was conducted as described previously (18). Rifampin resistance frequencies were also measured in the *E*. *coli ΔumuD772*::*kan* strain 315 carrying an expression vector pIX3.0 (Qiagen) bearing either *umuDAb* (pIX2), *umuDAbˊ* (pIX2*ˊ*), *umuD* (pIXUDEC), or no DNA insert. The *A*. *balylyi* ADP1 *umuDAb* gene was previously shown to be expressed from pIX2 in *E*. *coli* cells and to undergo self-cleavage in a RecA-dependent manner [20]. The *E*. *coli umuD* gene was amplified from strain AB1157 with primers shown in Table 2 and digested with *Bam*HI and *Xho*I for cloning into pIX3.0 according to the Qiagen EasyXpress Linear Template Kit Plus instructions.

### *I-TASSER* modelling

I-TASSER (Iterative Threading ASSEmbly Refinement) is a hierarchical method for protein structure and function prediction that identifies structural templates from the PDB by a multiple threading approach [23,24]. It then creates full-length atomic models in an iterative fashion through simulations. The modelling was performed by submission of a protein sequence to the online server accessible at: http://zhanglab.ccmb.med.umich.edu/I-TASSER/.

## Results

In testing whether UmuDAb regulates DNA damage-inducible gene expression by a LexA-type mechanism, we constructed N-terminal (NTD) and C-terminal domain (CTD) UmuDAb mutants by site-directed mutagenesis of a chromosomally-located *umuDAb* gene of *A*. *baylyi* ADP1. RT-qPCR experiments measured the expression of *umuDAb*-regulated DNA damage-inducible target genes such as *ddrR*, a gene of unknown function encoded adjacent to *umuDAb*, as well as *umuDAb* itself, to assess the effects of these mutations on UmuDAb-mediated gene repression and induction.

### A *umuDAb* null mutant expresses a sub-set of DNA damage-inducible genes at constitutively high levels

**Fig 1:**
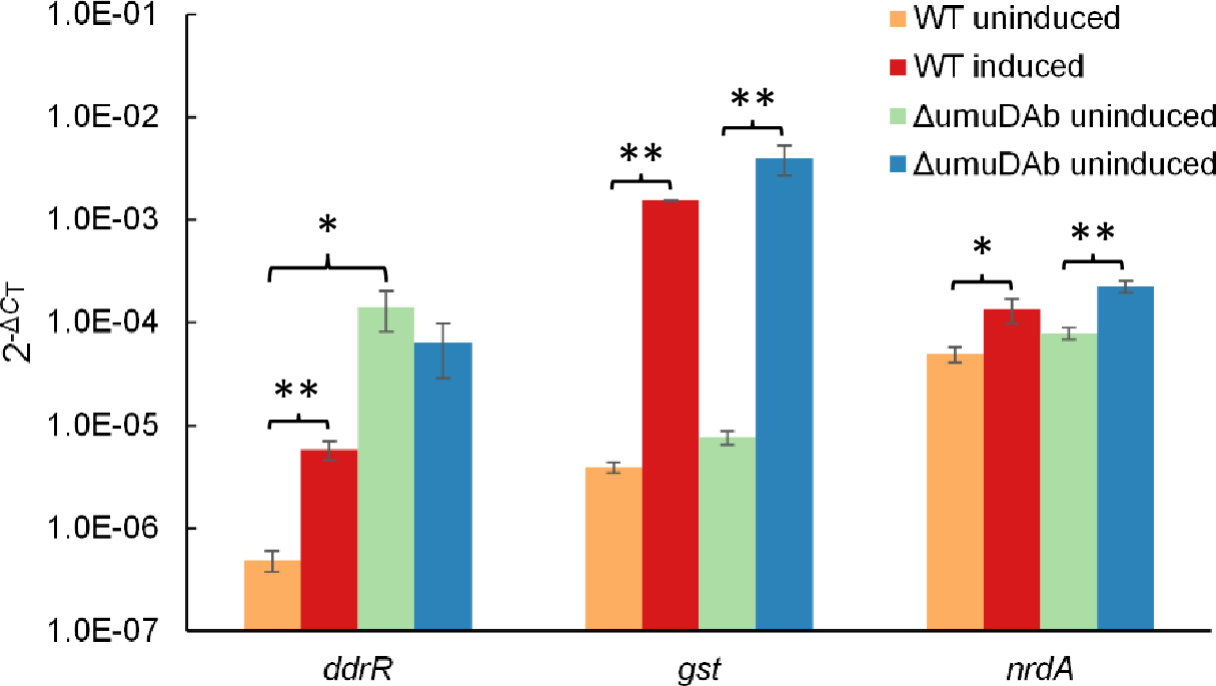
*A*. *baylyi* UmuDAb is required for repression of a sub-set of DNA damage-induced genes. RT-qPCR experiments measured expression of the *umuDAb*-regulated *ddrR* gene (*ACIAD2730*), and the non-*umuDAb* regulated genes *gst* (*ACIAD0445*) and *nrdA* (*ACIAD0724*) in the Δ*umuDAb* mutant strain ACIAD2729. The transcription of each gene was induced by DNA damage incurred by growth in 2 μg/mL MMC-containing medium. Each gene was assayed in one RT-qPCR experiment (plate), with error bars indicating standard error of the mean from technical triplicates of biological triplicates. Each gene was significantly induced in the wild type ADP1 strain (p < 0.05 designated by *; p < 0.01 designated by **). However, deletion of *umuDAb* (in ACIAD2729) resulted in a significant difference in the transcription of *ddrR* in MMC *vs* no MMC treatment (p < 0.01, as measured by 2^−ΔΔCT^), but not in the transcription of either *gst* or *nrdA* (p > 0.05).

We previously observed that in an *Acinetobacter baumannii* ATCC 17978 null *umuDAb* mutant, a sub-set of DNA damage-inducible genes were expressed at high levels (putatively, de-repressed) in the absence of DNA damage, specifically those related to error-prone, type V polymerase function such as *umuDAb* itself (*A1S_1389*), the *umuDC* homologs *A1S_2015*, *A1S_0636*, *A1S_1173* and *A1S_1174*, and a gene of unknown function encoded adjacent to *umuDAb*, *ddrR* (*A1S_1388*) [9]. Others have also observed similar regulation of these genes by UmuDAb [10]. However, *A*. *baylyi* ADP1 displays different patterns of regulation in its DNA damage-inducible genes than *A*. *baumannii* ATCC 17978, with some induced genes of ADP1 requiring neither *umuDAb* nor *recA* for their induction [9]. Furthermore, the ADP1 strain encodes no other error-prone type V polymerase genes besides *umuDAb* itself, suggesting that *umuDAb* may only repress itself and *ddrR* in *A*. *baylyi* ADP1. In the null *A*. *baylyi umuDAb* mutant strain ACIAD2729, *ddrR* expression was de-repressed in the absence of DNA damage, but the expression of DNA-damage inducible genes *gst* (*ACIAD0445*) and *nrdA* (*ACIAD0724*), which are not regulated by *umuDAb*, was still repressed in the absence of DNA damage (Fig 1).

### The NTD of UmuDAb is required for gene regulation activity

**Fig 2:**
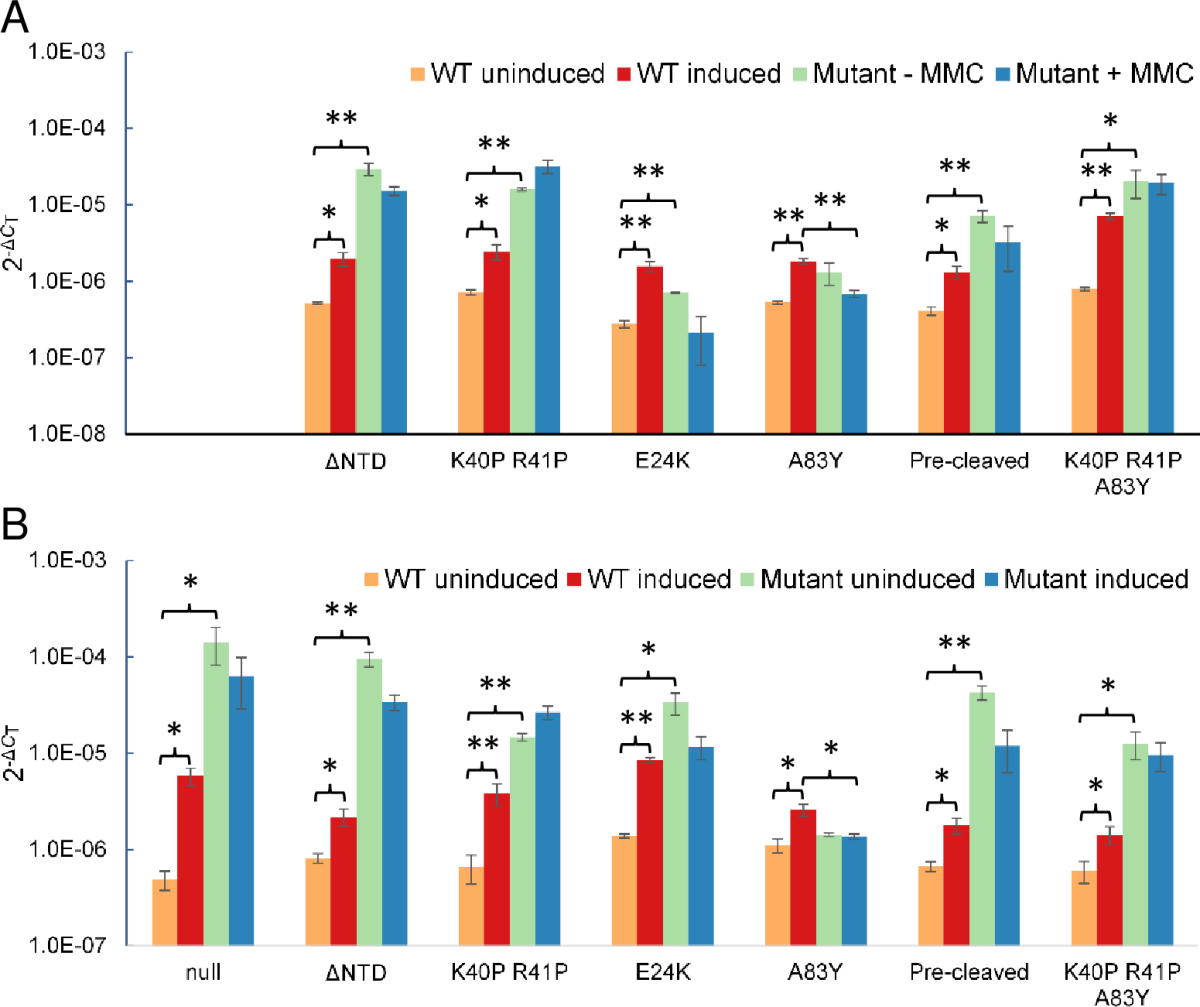
Regulation of DNA damage-inducible *ddrR* and *umuDAb* expression by UmuDAb requires NTD and CTD actions. RT-qPCR experiments measured gene expression, in the absence or presence of DNA damaging (2 μg/mL MMC) growth conditions, of (A) *umuDAb* (*ACIAD2729*) and (B) *ddrR* (*ACIAD2730*) in wild type ADP1 cells *vs* various *umuDAb* mutant strains). The specific type of mutant UmuDAb form is represented on the x-axes and comparable to each other in vertical alignment. Each gene was assayed in one RT-qPCR experiment (plate), with error bars indicating standard error of the mean from technical triplicates of biological triplicates. Statistical significance in a Student’s t-test is indicated by the symbol * for p values < 0.05, and by the symbol ** for p values < 0.01.

To identify the region within UmuDAb that contained repressor functionality for the regulation of both *umuDAb* and *ddrR*, we constructed ADP1 strain JHTW1 (*umuDAbΔ2–59*) whose UmuDAb protein does not encode the NTD that distinguishes the UmuDAb of *Acinetobacter* species [18] from the polymerase accessory protein UmuD. Like *umuD*, the mutant allele *umuDAbΔ2–59* only encodes the CTD preceded by 24 amino acids. We hypothesized that this ΔNTD UmuDAb protein would not be able to repress target gene expression in the absence of DNA damage, because the UmuD protein it now resembles has no transcriptional regulatory function. Expression of both *ddrR* and *umuDAb* in the absence of DNA damage was significantly higher (p < 0.01 in a one-tailed Student’s t-test) in this ΔNTD mutant than in wild type cells (Fig 2), and suggested that, as in LexA, the NTD of UmuDAb is required for target gene repression.

### Mutation of a potential DNA binding motif relieves target gene repression

**Fig 3:**
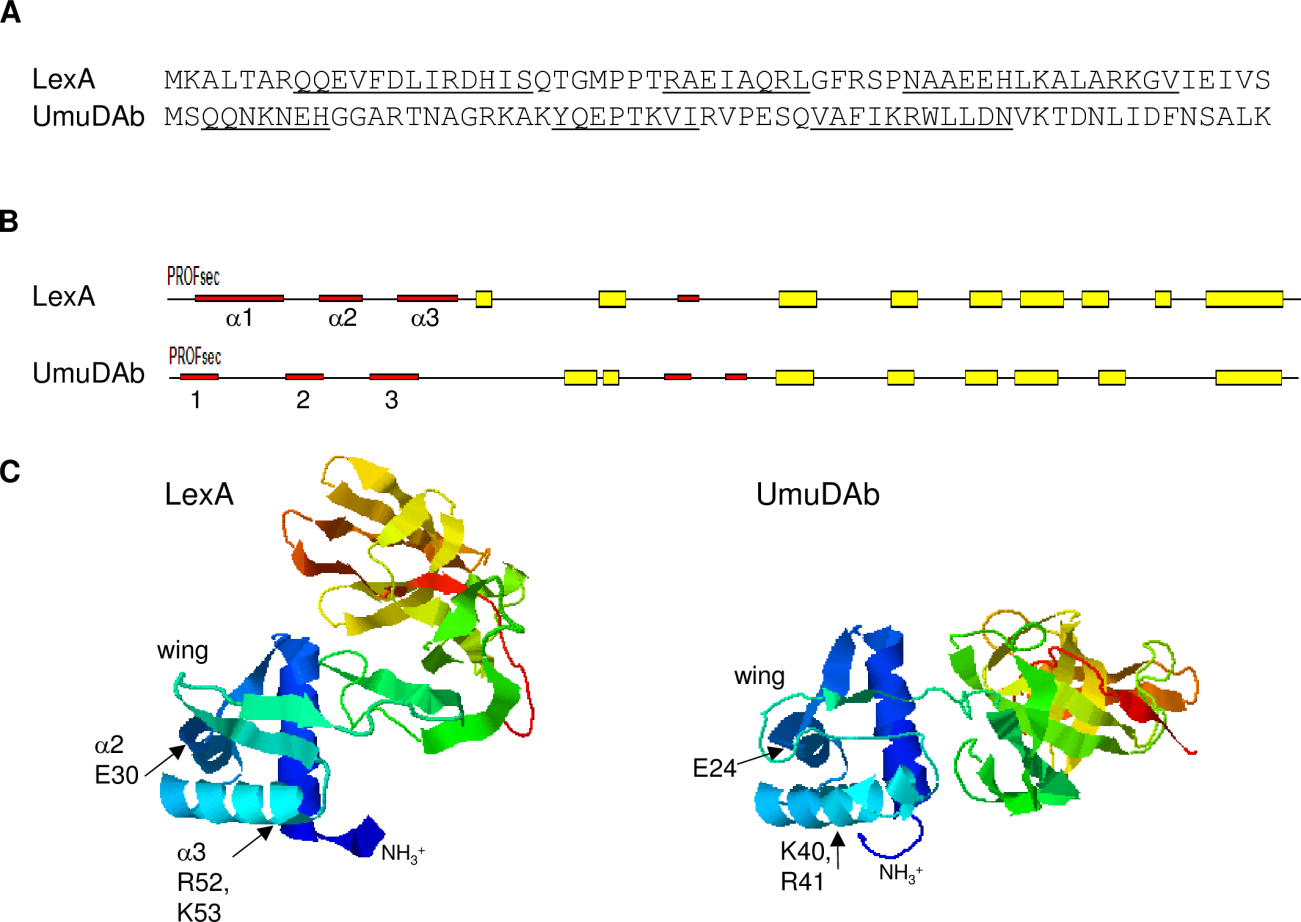
Modeling of the N-terminal domains of LexA and UmuDAb monomers. (A) The N-terminal 60 amino acids of *E*. *coli* LexA and *A*. *baylyi* UmuDAb, showing underlined alpha-helical regions predicted by the Predict Protein server [28]. In LexA, helices α1–3 span amino acids 8–20, 28–35, and 41–55 [25]. For UmuDAb, helices are predicted to form from amino acids 3–9, 22–29, and 36–46. (B) Predicted secondary structures of LexA and UmuDAb, showing alpha-helices 1–3 represented by red thin bars and beta sheets represented by thick yellow boxes; predicted by the Predict Protein server. (C) I-TASSER modeling of LexA and UmuDAb, oriented to align the NTDs (in blue shading) and showing the wing of the wHTH structures. The interdomain linker between the LexA NTD and CTD is extremely flexible [4] and is likely responsible for the variation between the two proteins’ total orientations. Arrows point to some of the amino acids in the LexA α2 helix and α3 recognition helix that are required for DNA binding [4], and the similarly located sites of directed mutations in UmuDAb.

LexA binds to the major groove of the operator sequence of target promoters (SOS box) with the third of its three alpha helices, recognition helix α3 [4], located in an atypical wHTH structure found in its NTD [25,26]. But no HTH or other DNA binding motif has been found in UmuDAb using conserved domain searches with BLAST algorithms or Pfam [23]. Furthermore, the UmuDAb NTD has only 10% identity with the LexA NTD, although they share 37% identity in their CTDs that facilitate self-cleavage [18]. However, the Predict Protein server [27] predicted that UmuDAb possessed three alpha-helices in its NTD, in a similar arrangement as it predicted the LexA wHTH motif (Fig 3) previously observed by NMR [25].

The three-dimensional arrangement of these alpha-helices was examined with I-TASSER modeling. I-TASSER (Iterative Threading ASSEmbly Refinement) is a hierarchical method for protein structure and function prediction that identifies structural templates from the PDB by a multiple threading approach [23,24]. This modelling revealed an overall structure of a three helix-containing NTD separated from the CTD by a disordered loop, shared between UmuDAb and LexA (Fig 3). The top four structural PDB analogs that I-TASSER identified for UmuDAb were LexA proteins from *Thermatoga* and *Escherichia*.

In LexA, helices two and three form the wHTH motif crucial to DNA binding [4]. The positively charged R52 K53 amino acids located at the end of the α3 recognition helix are important for mediating this DNA binding [4], and mutations at this location cause a LexA-deficient phenotype [29] We therefore mutated the UmuDAb R41 and K40 amino acids that lie similarly within the predicted helix three of the UmuDAb NTD by substituting helix-breaking Pro residues for K40 and R41, which I-TASSER modeling predicted would disrupt this helix and the overall NTD structure. Mutation of these amino acids prevented *ddrR* as well as *umuDAb* repression, as expression of both genes in the absence of DNA damage was significantly higher (p < 0.01) in *umuDAb K40P R41P* mutant cells than in wild type cells (Fig 2). We also noted a significant (p < 0.05) (but smaller than in wild type cells) induction of both target genes’ expression in this mutant. Alpha helix 2 of LexA requires amino acid E30 [29] to assist in DNA binding, prompting us to construct a mutation (*E24K*) at the corresponding site in *umuDAb*. This mutation in JHMP1 cells also produced higher levels of *ddrR* and *umuDAb* expression in the absence of DNA damage (Fig 2). These data suggested that repressor activity of the UmuDAb NTD requires helix-forming amino acids similar to those required for LexA DNA binding, and suggests a similar mechanism of UmuDAb repression.

### A non-cleavable UmuDAb mutant prevents target gene induction

UmuDAb self-cleavage after DNA damage requires the amino acids A83-G84, similar to the self-cleavage sites of LexA (A84-G85; [29]) and UmuD (A24-G25 or C24-G25) [20]. To test whether UmuDAb self-cleavage is necessary for the induction of gene expression after DNA damage, we constructed strain JHDS1, which expressed a cleavage-deficient UmuDAb A83Y protein. This *umuDAb A83Y* mutation rendered *umuDAb* and *ddrR* expression unresponsive to DNA damage (uninducible), unlike in wild type cells, where these genes are significantly induced (Fig 2). In JHDS1 cells there was no significant difference (p > 0.05) between either of these genes’ expression in either the absence or presence of MMC, suggesting that UmuDAb cleavage is necessary for the induction of *umuDAb* and *ddrR* after DNA damage. By contrast, the induction of the non-*umuDAb* regulated DNA damage-inducible gene *gst* [9] was unaffected by this mutation and retained significant induction after MMC treatment (p < 0.01; S2 Fig).

### UmuDAbˊ cannot repress target gene expression

We previously showed that a cleaved UmuDAb (UmuDAb’) is produced after treatment of cells with either MMC- or UV-mediated DNA damage, and that this action is dependent on RecA [20]. Based on known LexA repressor action, we predicted that uncleaved UmuDAb was required for *umuDAb* and *ddrR* repression and that UmuDAb self-cleavage would cause its subsequent dissociation from the promoter of SOS genes and allow induced SOS gene expression. We constructed a mutant strain of ADP1 that expressed only UmuDAb’ (a pre-cleaved UmuDAb) and measured the MMC-inducible expression of *ddrR* and *umuDAbΔ2–83* in these mutant cells and wild type cells. As shown in Fig 2, the JHKW1 *umuDAbΔ2–83* strain failed to repress *ddrR* and *umuDAb* expression in the absence of DNA damage (p < 0.01 comparing expression of wild type *vs* JHKW1 cells), suggesting that UmuDAb self-cleavage relieves gene repression and allows induction of target genes. By contrast, the induction of the non-*umuDAb* regulated DNA damage-inducible gene *gst* [9] was unaffected by this mutation and retained significant induction after MMC treatment (p < 0.01; S2 Fig).

### Mutation of putative repressor binding sites in the *umuDAb*-*ddrR* promoter dysregulates *umuDAb* and *ddrR* expression

**Fig 4:**
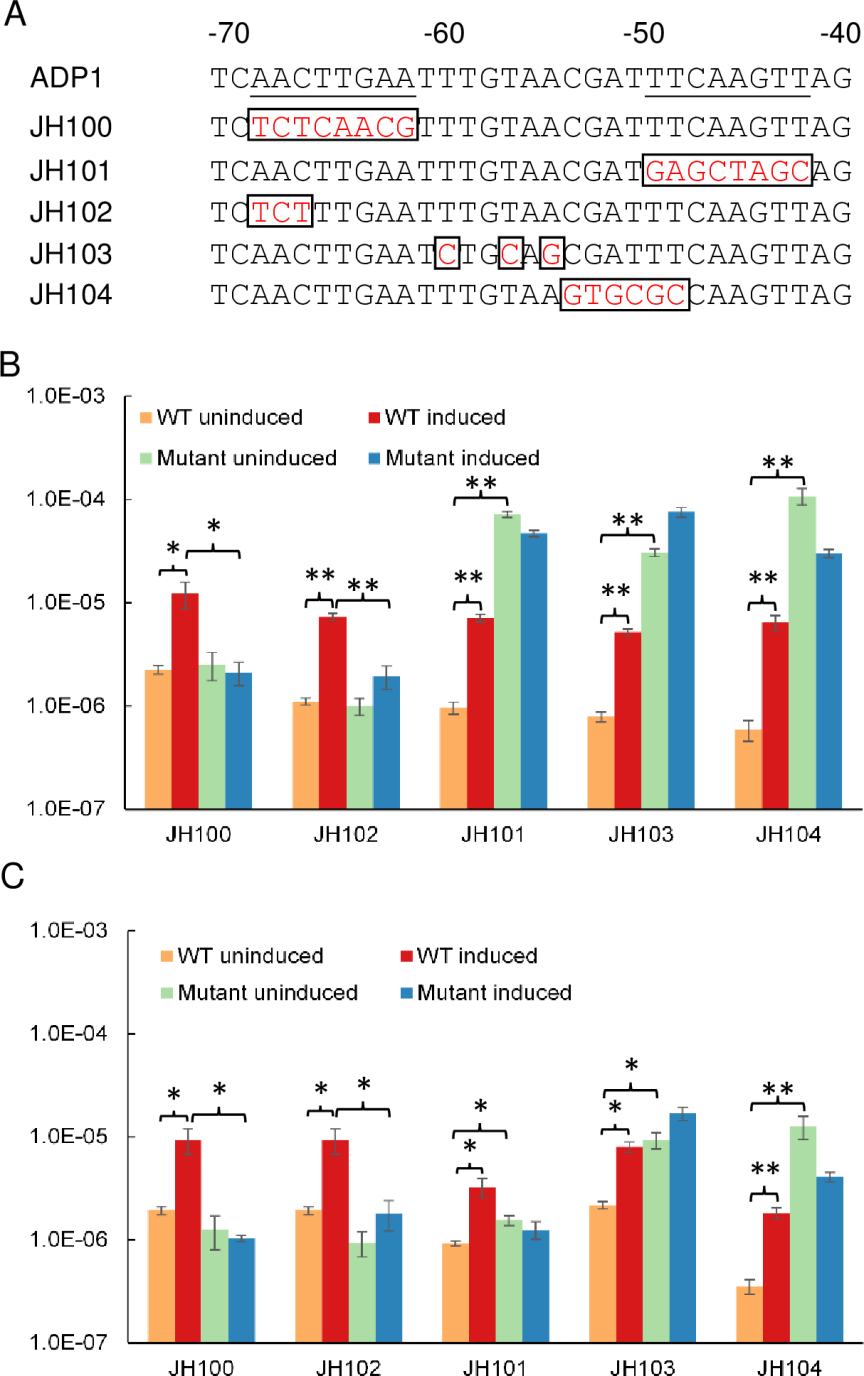
Mutation of potential operator site in *umuDAb*-*ddrR* promoter dysregulates UmuDAb-regulated gene expression. (A) Underlined nucleotides are those previously identified as possible regulatory protein binding sites (an SOS box) in ADP1, due to their palindromic nature [11]. The numbering system represents the number of nucleotides upstream of the *umuDAb* coding region. Nucleotides -66 through -45 (similar but non-identical in *A*. *baumannii*) were identified as required for UmuDAb binding to *A*. *baumannii* DNA fragments *in vitro* [10]. Mutations in ADP1 mutant strains JH100-104 are represented in red boxes. RT-qPCR experiments measured *ddrR* (B) and *umuDAb* (C) expression in uninduced or induced (2 μg/mL MMC) wild type ADP1 *vs* mutant cells. Each gene was assayed in one RT-qPCR experiment (plate), with error bars indicating standard error of the mean from technical triplicates of biological triplicates. Statistical significance in a Student’s t-test is indicated by the symbol * for p values < 0.05, and by the symbol ** for p values < 0.01.

We previously identified a palindromic sequence in the putative *umuDAb-ddrR* promoter region of ADP1 (AACTTGAA(N_11_)TTCAAGTT) that might be a regulatory protein binding site [11]. Aranda *et al*. showed that purified UmuDAb of *A*. *baumannii* ATCC 17978 binds to a very similar region in the *umuDAb-ddrR* promoter of this species, and in the promoters of the *umuDC* operons *A1S_0636–0637*, *A1S_1173–1174* (and *umuC* homologs *A1S_2008* and *A1S_2015*) [10]. However, experiments were not performed to test whether these sequences are required for repression (or induction) of these genes. We addressed this issue by constructing several mutant strains (JH100, JH101, JH102 and JH104) with site-directed mutagenesis to contain various mutations in either of the predicted DNA binding sites (half-site operator) of the *umuDAb*-*ddrR* promoter, as well as the base pairs between these half-sites (JH103). These mutations, shown in Fig 4A, disrupted the palindromic nature of the motif by replacing the wild-type nucleotides with non-palindrome-forming nucleotides (except for in JH103, where two of the three changed nucleotides preserved their palindromic nature). The expression of both *umuDAb* and *ddrR* in the absence of DNA damage was significantly increased in the strains JH101, JH103, and JH104, relative to their expression in wild type cells (Fig 4B), suggesting that this motif is required for target gene repression. However, mutation of the more distal (from the *umuDAb* open reading frame) half-site in strains JH100 and JH102 did not affect transcription in the uninduced condition, but rather prevented induction of transcription after DNA damage. As a putative -35 promoter consensus element is located adjacent to this distal half-site [11], it is possible that the mutation constructed at this site affected RNA polymerase recognition of the consensus element and prevented transcription in both conditions, as we observed.

### Constructing a model of UmuDAb repression and induction mechanisms

In order to evaluate the order of UmuDAb actions in the regulation of *umuDAb*-*ddrR*, we constructed the triple mutant strain JHDT1 (*umuDAb K40P R41P A83Y*) that combined the putative HTH mutation (*umuDAb K40P R41P*) with the non-cleavable *umuDAb A83Y* mutation. We predicted that this triple mutant would be unable to repress gene expression in the absence of DNA damage, due to disruption of the putative HTH region in the NTD. Furthermore, we hypothesized that JHDT1 cells would lack even the small remaining amount of target gene induction after DNA damage seen in the JHTW2 *umuDAb K40P R41P* strain (Fig 2), due to addition of the non-cleavable *umuDAbA83Y* mutation.

We evaluated the triple mutant’s regulatory activity and observed it to be consistent with this model. As shown in Fig 2, expression of *umuDAb* and *ddrR* in the absence of DNA damage was higher in JHDT1 cells than in wild type cells (p < 0.01). Additionally, direct comparison between JHDT1 and the HTH mutant strain JHTW1 in RT-qPCR expression experiments showed that the small but significant amount of induction observed in the HTH mutant was absent in the triple mutant (p < 0.05). This experiment suggested that the UmuDAb K40P R41P amino acids are required for initial repression, and that the repressed state was released upon UmuDAb self-cleavage, which was prevented by the A83Y mutation.

### The UmuDAb homolog does not provide a UmuD type V polymerase accessory function

**Fig 5:**
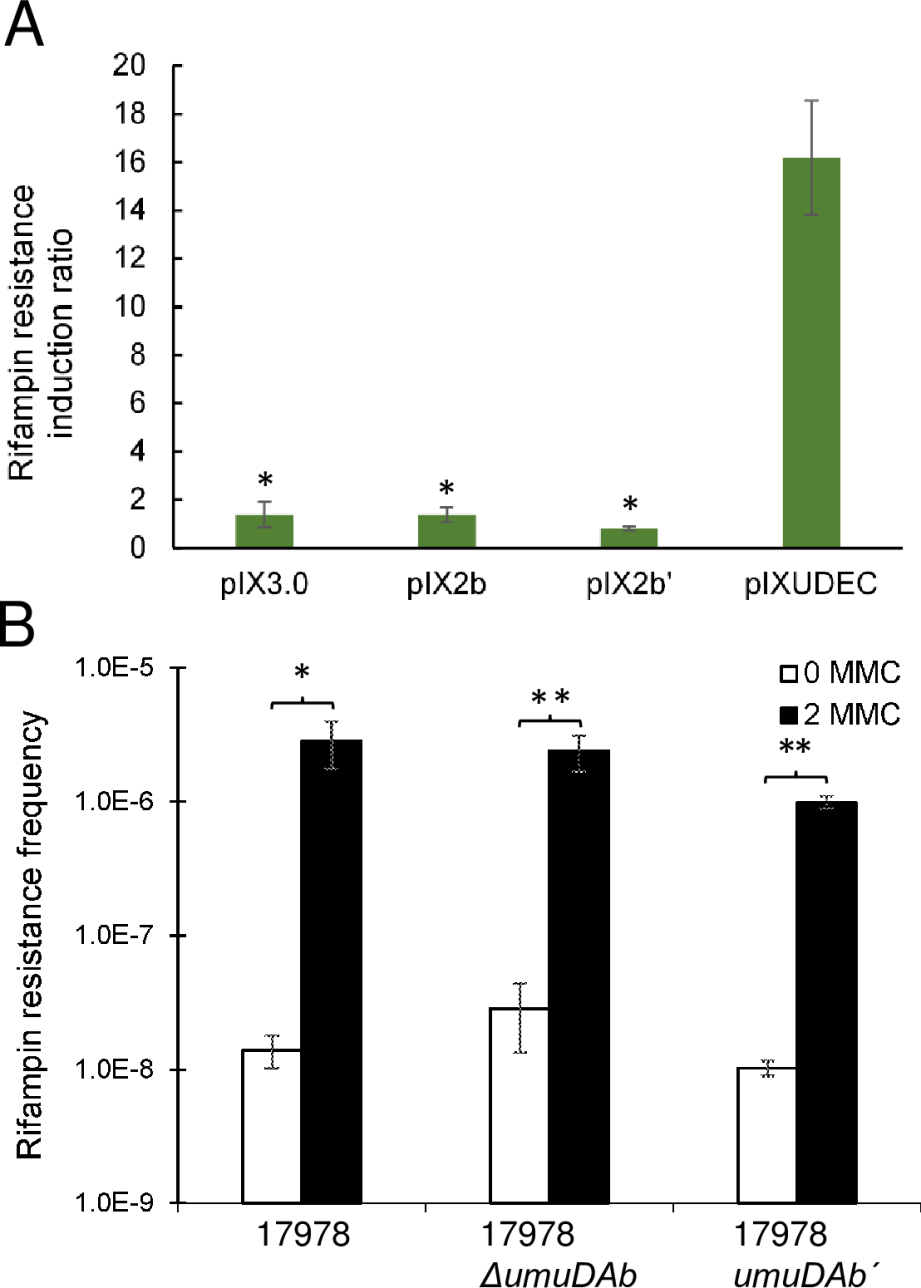
DNA damage-induced mutagenesis experiments suggest that UmuDAb does not perform UmuD polymerase accessory function. (A) DNA damage-induced mutagenesis (measured by, and represented as, the ratio of increased rifampin resistance observed in UV-treated *vs* untreated cells) was performed to assess whether *umuDAb* could complement an *E*. *coli ΔumuD* mutant. The *E*. *coli* Δ*umuD772*::kan strain 315 carried either: pIX3.0 (containing no DNA insert), pIX2b (pIX3.0 carrying *A*. *baylyi umuDAb* [20]), pIX2bˊ (pIX3.0 carrying *A*. *baylyi umuDAb*ˊ (*umuDAbΔ2–83*), or pIXUDEC (pIX3.0 carrying *E*. *coli umuD*). Differences among strains were analyzed with a two-tailed, one-way Kruskal-Wallis ANOVA, followed by Dunn’s multiple comparisons post-test indicating significance denoted by * (p < 0.05 when compared to the vector carrying *E*. *coli umuD*). (B) No reduction in the induced rifampin resistance was observed in the presence of DNA damage in *A*. *baumannii* Δ*umuDAb* or *umuDAb*ˊ (*umuDAbΔ2–83*) cells, relative to wild type cells (p < 0.05). Data are represented as the average of a minimum of four (panel A) or six (panel B) experiments with error bars representing the standard error of the mean.

The UmuD encoded by a *umuDC* operon composes part of the type V polymerase UmuDˊ_2_C that carries out translesion, error-prone DNA synthesis (SOS mutagenesis) after DNA damage. Previous work expressing UmuDAb in *E*. *coli* and demonstrating its self-cleavage by *E*. *coli* RecA after DNA damage [20] allowed us to attempt complementation of an *E*. *coli umuD* mutant with various wild-type and mutant *Acinetobacter umuDAb* alleles. In an SOS mutagenesis assay, the ADP1 *umuDAb* gene could not complement an AB1157 Δ*umuD772*::kan mutant (Fig 5A). Neither a plasmid carrying *umuDAb* nor a plasmid carrying *umuDAbˊ* provided a significantly different induction of rifampin resistant mutants than did the empty vector construct (p < 0.05 in a Kruskal-Wallis ANOVA). Complementation of this Δ*umuD772* mutant with the *E*. *coli umuD* allele, however, yielded induction of rifampin resistance at a significantly higher level (~16-fold increase in rifampin resistant mutants; Fig 5A).

We also evaluated the frequency of *A*. *baumannii* DNA damage-induced mutagenesis in both a *umuDAb* null mutant and a *umuDAb*ˊ (*umuDAbΔ2–83*) mutant as compared to wild type cells (Fig 5B). We observed that the induced level of mutation to rifampin resistance was not significantly different in the 17978 Δ*umuDAb* mutant *vs* wild type cells (p > 0.05). The background level of mutation to rifampin resistance was slightly higher in the *umuDAb* null mutant than in wild type cells, but this was not statistically significant (p > 0.05). These data do not suggest that UmuDAb is required for error-prone polymerase activity like the UmuD accessory [9], but are consistent with the model that UmuDAb performs regulatory functions.

## Discussion

These experiments have demonstrated that the actions of UmuDAb in regulating gene expression after DNA damage are consistent with a LexA-type repressor mechanism: repression mediated through the NTD being relieved by cleavage in the CTD. In this study, we identified a putative helix-turn-helix (HTH) motif in the NTD of UmuDAb that is similar to the wHTH structure with which LexA binds to promoters and represses DNA damage-induced genes (Fig 3). In *umuDAb* the presumed start codon (Met) is preceded by five additional codons in an open reading frame, beginning with a Val codon, which is an alternate start codon for bacteria. It is unknown whether UmuDAb contains these additional five amino acids as its N-terminus, but addition of these amino acids would create a nearly perfect alignment of the NTD helices’ placement in UmuDAb and LexA (S3 Fig) that might even better explain the action we observed for the NTD. I-TASSER modeling did not predict any structural difference in the NTD of UmuDAb if these five amino acids were part of the overall protein structure.

This NTD model allowed us to identify amino acids K40 R41 as being similarly placed and positively charged amino acids in each protein’s helix 3 (in LexA, the α3 recognition helix). We mutated these residues to disrupt the potential structure, and possible DNA binding, of this helix. Promoter binding activity has been observed *in vitro* for the UmuDAb protein of *A*. *baumannii* ATCC 17978 [10], which is 79% identical to the *A*. *baylyi* ADP1 UmuDAb and possesses essentially the same structure in I-TASSER modeling, and so may point to a shared DNA binding activity for both proteins. Mutation of one (*E24K*) or two (*K40P R41P*) charged amino acids in either of these two potential helical regions in the NTD of UmuDAb results in the de-repression of the *umuDAb* and *ddrR* target genes in the absence of DNA damage, identifying these amino acids as playing a significant role in the repression mediated by UmuDAb.

A detailed examination of the K40P R41P mutant reveals that, while the helix-containing NTD domain is necessary to provide a significant component of the repressive action of UmuDAb, the K40 R41 amino acids are not likely the only residues needed to confer this repression by the NTD. For example, the level of *ddrR* expression in the absence of DNA damage is significantly less (p < 0.05) in the K40P R41P mutant than in either the NTD or UmuDAb null mutants. Similarly, mutations in the LexA recognition helix α3 that binds to the DNA in the major groove are insufficient to totally de-repress gene expression [4]. Mutations within LexA helix 2, as well as its wing, also de-repress gene expression, indicating that multiple NTD regions play a role in stabilizing the interaction of LexA and DNA [4]. It is expected that other elements of the UmuDAb HTH structure may also contribute to a stable repressor-DNA interaction.

Examination of target gene expression in the UmuDAb K40P R41P A83Y triple mutant suggests that the interference in the predicted helix 3 of the NTD that prevents gene repression, precedes the DNA damage-induced UmuDAb self-cleavage, so the cleaved state is irrelevant for overall UmuDAb function while in the K40P R41P mutant form.

We have also investigated the extent and role in gene expression of the inverted repeats present in the shared ADP1 *ddrR*-*umuDAb* promoter region [11]. Mutation of this putative (for *A*. *baylyi*) UmuDAb binding site in the abrogates repression of these target genes’ expression in the absence of DNA damage in strains JH101, JH103 and JH104, where the more proximal (to *umuDAb*) half operator site was mutated. The mutation in strain JH104 was designed to mutate part of the inverted repeat we observed in the ADP1 strain [11] (entirely mutated in JH101), as well as a less-conserved region required for UmuDAb binding *in vitro* in *A*. *baumannii* [10]. In mutant strain JH103, the mutation was made not in either of the inverted repeats we had identified, but in the central spacer region separating them. Interestingly, although two of the three mutations in this strain, A(-54)G and T(-56)C, preserved the palindromic nature in the spacer region, gene expression was disrupted similarly to the JH101 and JH104 mutant strains. This suggested that not only the inverted repeats themselves, but the spacer region, and more specifically, the actual identity of the nucleotides in the spacer, were important for repression. This is not a feature of other SOS boxes. The *A*. *baumannii* UmuDAb binds to a very similar set of nucleotides in the *A*. *baumannii ddrR*-*umuDAb* promoter region [10], where changing the same A(-54) and T(-56) nucleotides to a C and G, which similarly preserved the palindromic nature of this area, likewise disrupted DNA binding. Those data support the hypothesis that the repression of gene expression is mediated through UmuDAb binding to DNA. The qualitatively equivalent de-repression pattern observed for both *ddrR* and *umuDAb* expression both before and after DNA damage (Fig 4) suggests that the mutation we constructed in the putative promoter region shared by both genes affects a common mechanism of repression.

The two mutant strains whose mutations were placed in the half-operator site more distal to *umuDAb* (nucleotides -68 through -61 in Fig 4A; JH100 and JH102), demonstrated a lack of induction of gene expression after DNA damage, suggesting a disruption in overall transcription. This possibility is supported by the observation that in JH102, gene expression is disrupted even though the analogous (and identical) base pairs in *A*. *baumannii* are not required for UmuDAb binding to DNA *in vitro* [10].

A previous study in *A*. *baumannii* ATCC 17978 reported a significant decrease in the rifampin resistance mutation frequency of a *umuDAb* mutant upon UV treatment, ascribing error-prone polymerase accessory function to UmuDAb, but this mutant was not a null *umuDAb* mutant [19], as in this study, but rather a disruption mutant that left a partially intact (truncated in its CTD) *umuDAb* coding region, which might encode a dominant negative UmuDAb repressor. However, LexA requires its CTD for the dimerization state that helps it bind promoters. When its self-cleavage separates the CTD from the DNA-binding NTD, its DNA-binding is weakened [30]. This model therefore suggests that a CTD-truncated UmuDAb protein might not be able to effect repression, although it is not known whether UmuDAb forms dimers, requires dimerization for its DNA binding, or whether its CTD is required in such putative dimerization. Our observation that *umuDAb* did not complement an *E*. *coli umuD* mutant, is also most consistent with UmuDAb functions separate from error-prone polymerase activity.

**Fig 6:**
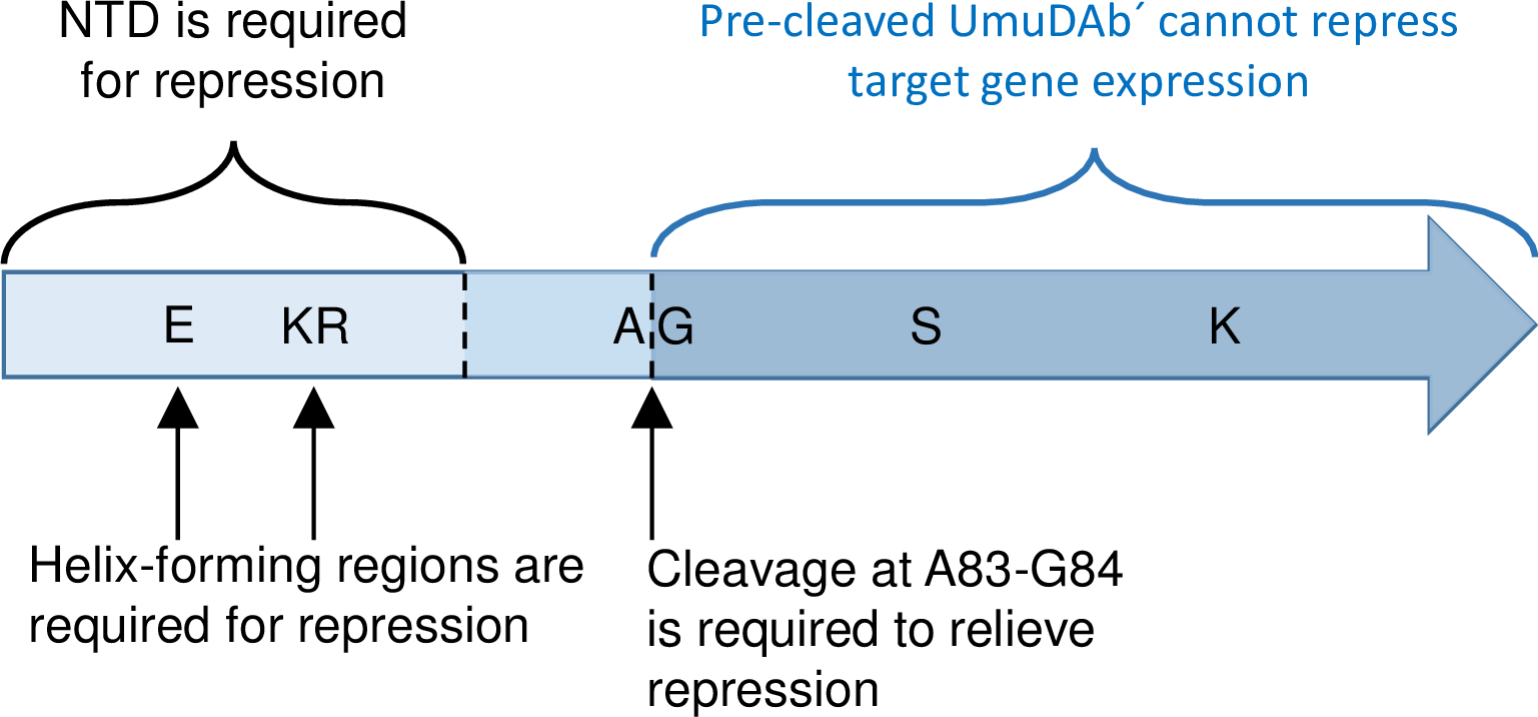
Model of amino acid motifs and domains required for UmuDAb-mediated gene repression and induction. Results of gene expression studies with defined *umuDAb* mutants revealed specific amino acid motifs and protein regions required for repression (NTD) and induction (CTD) of DNA damage-inducible genes. The roles of A83-G84, S119 and K156 were established for UmuDAb self-cleavage previously [20].

We propose that UmuDAb is part of a regulatory system that functions in *Acinetobacter* like LexA—to regulate the expression of a sub-set of DNA damage-inducible genes. Genes typically involved in DNA repair functions (such as *dnaN*, *ruvA*, *uvrC*, *recN*, *recG*, *dnaQ*) are not induced by DNA damage in *A*. *baylyi* and/or *A*. *baumannii* ATCC 17978 [9,10]. However, a subset of DNA damage responsive genes, specifically those encoding error-prone polymerase subunits, are both induced upon DNA damage as well as regulated (in a manner suggestive of repression) by UmuDAb. Similarly supporting the specialized role of UmuDAb repression in this genus is the observation that the expression of *recA*, although induced by DNA damage, is unaffected by the loss of UmuDAb (or, surprisingly, itself [13]). Overall, these experiments suggest that UmuDAb repression is accomplished via the putative HTH motif in its NTD, to target promoters in the absence of DNA damage. The subsequent relief of that repression when DNA damage triggers UmuDAb self-cleavage at its known A83 G84 cleavage site in its CTD [20] then allows induction of transcription (Fig 6). The specialized target regulon of UmuDAb also suggests that it might be possible to pursue strategies to inhibit the autocatalytic cleavage of UmuDAb to prevent the expression of error-prone polymerase genes that produce antibiotic resistance in the opportunistic, often multi-drug resistant pathogen *A*. *baumannii*.

## Supporting Information

S1 Fig Western analyses show expression of mutant UmuDAb proteins. The panels depict the expression of UmuDAb from wild type and mutant *umuDAb* alleles found in: (A) wild type (WT), JHKW1 (expressing UmuDAbˊ; UDˊ), and JHTW1 (ΔNTD), (B) JHTW2 (K40P R41P) and JHDS1 (A83Y), and (C) JHMP1 (E24K), JHDT1 (K40P R41P A83Y), and JHDS1 (A83Y) strains of *A*. *baylyi* ADP1. The right side of panel C, again showing JHMP1 and JHDT1, with four messy lanes included to demonstrate the smiling affecting the position of UmuDAb on the left *vs* right side of the gel. Expression of these mutant proteins was detected as described previously [20], with the sizes of protein standards (Precision Plus Protein WesternC Protein Standards) shown in kD and designated by lane label “M”. Plus and minus signs for the strain JHTW2 indicate whether treatment with 2 μg/mL MMC was present. Treatment of JHTW2 cells resulted in cleavage of UmuDAb, as the *umuDAb K40P R41P* allele was not predicted to affect either the A83-G84 cleavage sites residues, or the CTD catalytic domain of the protein.
(PPTX)

S2 Fig Expression of *gst* (ACIAD0445) is not affected by mutations in *umuDAb*. The expression of *gst* was measured in induced (2 μg/mL MMC) or uninduced cells and is reported as 2^-C^_T_ levels on the y-axis. All *A*. *baylyi* ADP1 strains, carrying either a wild type (ADP1) or mutant *umuDAb* allele, had significantly increased *gst* expression after MMC induction (p < 0.01 in a t-test of each strain), but were not different in their induction levels (p > 0.05 in a Kruskal-Wallis ANOVA).
(TIF)

S3 Fig Alignment of predicted alpha helices in the N-terminal portion of UmuDAb and LexA. The *A*. *baylyi umuDAb* open reading frame contains five additional codons preceding the Met start codon. As the first codon is GTG (encoding valine), an alternate start codon in bacteria, it is possible that the UmuDAb protein contains these amino acids. Inclusion of these five amino acids results in a better linear alignment of the alpha helical regions of these proteins
(PPTX)

S1 Table Efficiencies of primers used in RT-qPCR experiments. Primer efficiencies were calculated over five orders of magnitude of template (diluted genomic DNA from *A*. *baylyi* ADP1), as recommended [22].
(DOCX)
