# Supplementary figures and images for: UmuDAb: An Error-Prone Polymerase Accessory Homolog Whose N-Terminal Domain Is Required for Repression of DNA Damage Inducible Gene Expression in Acinetobacter baylyi
Source: PLoS One. 2016 Mar 24;11(3):e0152013. doi: 10.1371/journal.pone.0152013 (PMC4807011; doi:10.1371/journal.pone.0152013)

## Slide 1
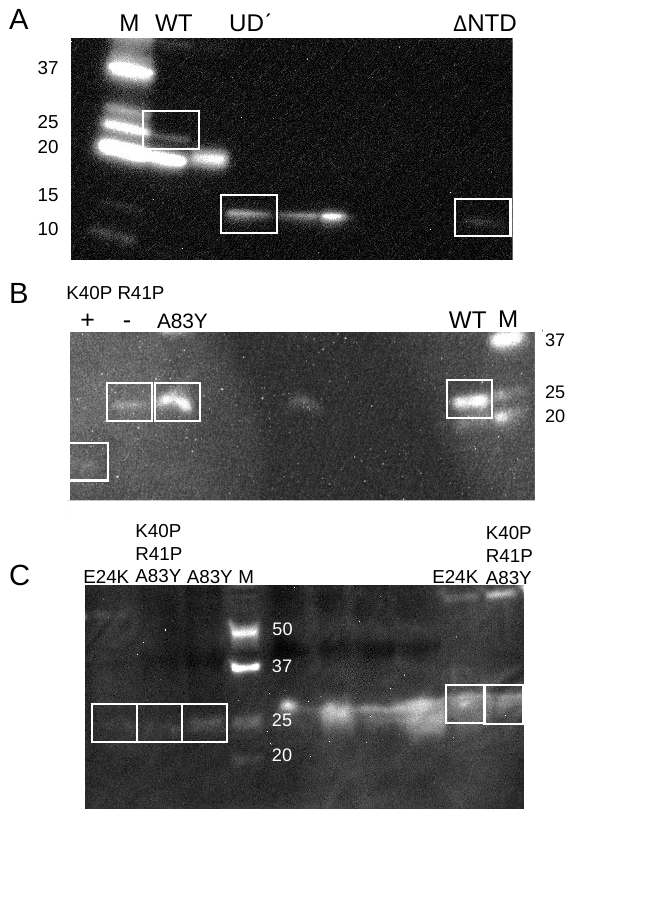

A
M
WT
UDˊ
ΔNTD
37
25
20
15
10
B
K40P R41P
 + -
M
WT
A83Y
37
25
20
K40P R41P
A83Y
K40P R41P
A83Y
C
E24K
A83Y
E24K
M
50
37
25
20

Supplement: S1 Fig — The panels depict the expression of UmuDAb from wild type and mutant umuDAb alleles found in: (A) wild type (WT), JHKW1 (expressing UmuDAbˊ; UDˊ), and JHTW1 (ΔNTD), (B) JHTW2 (K40P R41P) and JHDS1 (A83Y), and (C) JHMP1 (E24K), JHDT1 (K40P R41P A83Y), and JHDS1 (A83Y) strains of A. baylyi ADP1. The right side of panel C, again showing JHMP1 and JHDT1, with four messy lanes included to demonstrate the smiling affecting the position of UmuDAb on the left vs right side of the gel. Expression of these mutant proteins was detected as described previously [20], with the sizes of protein standards (Precision Plus Protein WesternC Protein Standards) shown in kD and designated by lane label “M”. Plus and minus signs for the strain JHTW2 indicate whether treatment with 2 μg/mL MMC was present. Treatment of JHTW2 cells resulted in cleavage of UmuDAb, as the umuDAb K40P R41P allele was not predicted to affect either the A83-G84 cleavage sites residues, or the CTD catalytic domain of the protein. (PPTX) [file pone.0152013.s001.pptx]
