# Supplementary material for: UmuDAb: An Error-Prone Polymerase Accessory Homolog Whose N-Terminal Domain Is Required for Repression of DNA Damage Inducible Gene Expression in Acinetobacter baylyi
Source: PLoS One. 2016 Mar 24;11(3):e0152013. doi: 10.1371/journal.pone.0152013 (PMC4807011; doi:10.1371/journal.pone.0152013)
Supplement: S3 Fig — The A. baylyi umuDAb open reading frame contains five additional codons preceding the Met start codon. As the first codon is GTG (encoding valine), an alternate start codon in bacteria, it is possible that the UmuDAb protein contains these amino acids. Inclusion of these five amino acids results in a better linear alignment of the alpha helical regions of these proteins (PPTX) [file pone.0152013.s003.pptx]

## Slide 1
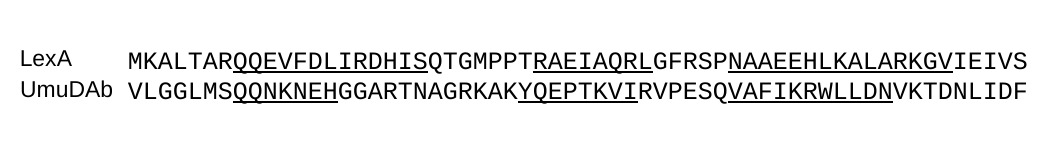

LexA
MKALTARQQEVFDLIRDHISQTGMPPTRAEIAQRLGFRSPNAAEEHLKALARKGVIEIVS
VLGGLMSQQNKNEHGGARTNAGRKAKYQEPTKVIRVPESQVAFIKRWLLDNVKTDNLIDF
UmuDAb
