# Supplementary material for: UmuDAb: An Error-Prone Polymerase Accessory Homolog Whose N-Terminal Domain Is Required for Repression of DNA Damage Inducible Gene Expression in Acinetobacter baylyi
Source: PLoS One. 2016 Mar 24;11(3):e0152013. doi: 10.1371/journal.pone.0152013 (PMC4807011; doi:10.1371/journal.pone.0152013)
Supplement: S1 Table — Primer efficiencies were calculated over five orders of magnitude of template (diluted genomic DNA from A. baylyi ADP1), as recommended [22]. (DOCX) [file pone.0152013.s004.docx]

**S1 Table.** Efficiencies of primers used in RT-qPCR experiments.

| Gene amplified by primer set | Efficiency (%) |
| --- | --- |
| 16S rRNA | 94.5 |
| *ddrR* (*ACIAD2730*) | 94.4 |
| *umuDAb* (*ACIAD2729*) | 93.1 |
| *gst* (*ACIAD0445*) | 91.6 |
| *nrdA* (*ACIAD0724*) | 93.6 |
